# Coordinated regulation of hepatic and adipose tissue transcriptomes by the oral administration of an amino acid mixture simulating the larval saliva of *Vespa* species

**DOI:** 10.1186/s12263-016-0534-2

**Published:** 2016-07-11

**Authors:** Fumika Shinozaki, Takashi Abe, Asuka Kamei, Yuki Watanabe, Akihito Yasuoka, Kosuke Shimada, Kaori Kondo, Soichi Arai, Kota Kumagai, Takashi Kondo, Keiko Abe

**Affiliations:** 1 Project for Development of Food Functionality Assessment Methods, Kanagawa Academy of Science and Technology, Life Science & Environment Research Center (LiSE) 4F C-4, 3-25-13 Tonomachi, Kawasaki-ku, Kawasaki, Kanagawa 210-0821 Japan; 2 Hornet Research Center, 1-48-7-201 Matsubara, Setagaya-Ku, Tokyo, 156-0043 Japan; 3 NODAI Research Institute, Tokyo University of Agriculture, 1-1-1 Sakuragaoka, Setagaya-ku, Tokyo, 156-8502 Japan; 4 KYOWA HAKKO BIO CO., LTD., 2, Miyukigaoka, Tsukuba, Ibaraki 305-0841 Japan; 5 Department of Applied Biological Chemistry, Graduate School of Agricultural and Life Sciences, The University of Tokyo, 1-1-1 Yayoi, Bunkyo-ku, Tokyo, 113-8657 Japan

**Keywords:** Gene expression, Amino acid mixture, Liver, DNA microarray, Adipose tissue

## Abstract

**Background:**

VAAM is an amino acid mixture that simulates the composition of *Vespa* larval saliva. VAAM enhanced physical endurance of mice and have been used by athletes as a supplementary drink before exercise. However, there is no information on the effect of VAAM on the physiology of freely moving animals. The purpose of this study was to obtain information about the VAAM-dependent regulation of liver and adipose tissue transcriptomes.

**Results:**

Mice were orally fed a VAAM solution, an amino acid mixture mimicking casein hydrolysate (CAAM) or water under ad libitum feeding conditions for 5 days. Comparisons of the hepatic transcriptome between VAAM-, CAAM-, and water-treated groups revealed a VAAM-specific regulation of the metabolic pathway, i.e., the down-regulation of glycolysis and fatty acid oxidation and the up-regulation of polyunsaturated fatty acid synthesis and glucogenic amino acid utilization. Similar transcriptomic analyses of white and brown adipose tissues (WAT and BAT, respectively) indicated the up-regulation of phospholipid synthesis in WAT and the negative regulation of cellular processes in BAT. Because the coordinated regulation of tissue transcriptomes implied the presence of upstream signaling common to these tissues, we conducted an Ingenuity Pathways Analysis. This analysis showed that estrogenic and glucagon signals were activated in the liver and WAT and that beta-adrenergic signaling was activated in all three tissues.

**Conclusions:**

We found that VAAM ingestion had an effect on multiple tissue transcriptomes of freely moving mice. Utilization of glycogenic amino acids may have been activated in the liver. Fatty acid conversion into phospholipid, not to triacylglycerol, may have been stimulated in adipocytes contrasting that a little effect was observed in BAT. Analysis of upstream factors revealed that multiple hormonal signals were activated in the liver, WAT, and BAT. Our data provide some clues to understanding the role of VAAM in metabolic regulation.

**Electronic supplementary material:**

The online version of this article (doi:10.1186/s12263-016-0534-2) contains supplementary material, which is available to authorized users.

## Background

The amino acid composition of food has a large impact on the metabolic homeostasis of individuals, especially when they are supplied as a major nutrient. As clearly understandable by the polyribosome model of protein synthesis, a shortage of the first-limiting amino acids readily affects the supply of proteins as the source of enzymes and other tissue-constructing materials. In this regard, essential amino acids (EAAs) are critical for the growth of the animal owing to their inability to synthesize EAAs from an organic backbone and nitrogen donor. In addition, many amino acids possess organic acid moieties that can serve as substrates for energy production (glucogenic and ketogenic amino acids).

In addition to these nutritive functions of amino acids, some dietary amino acids play roles as extracellular signals. Branched chain amino acids (BCAAs) constitute approximately 50 % of dietary EAAs and are known to activate protein anabolism via the mammalian target of rapamycin (mTOR)-dependent pathway [1, 2]. Glycine, glutamate, phenylalanine, histidine, and tryptophan act as or are metabolized to neurotransmitters and thus are able to exert a modulatory effect on the nervous system [3]. Portions of gastrointestinal epithelial cells express l-amino acid-sensing taste receptors and stimulate gastrointestinal hormone secretion in response to phenylalanine, leucine, glutamate, and tryptophan administration [4, 5]. These non-nutritive functions are attributable to a small number of amino acids but not to their whole constituent amino acids.

We have investigated the regulatory role of an amino acid mixture that simulates *Vespa* species (VAAM) on motor performance in animals at the dosing condition of sub-nutritional level (0.38 % of total food-derived energy/day). VAAM consists of 17 amino acids, among which glycine, tryptophan, and proline are present at an approximately twofold higher molar ratio than diet-derived amino acids (Additional file 1: Table S1). We first searched the optimum condition of VAAM administration to achieve significant enhancement of exercise performance [6]. In one study, a preceding oral administration of VAAM at the dose of 0.67 g/kg body weight improved the swimming performance of mice; this improvement was not observed with an amino acid mixture simulating the amino acid composition of bovine casein (CAAM) [7]. In addition, VAAM can lower serum lactate levels and activate fatty acid release into mice sera and into the media of cultured rat adipocytes [6, 8]. VAAM solution, as a sport supplement drink, has been in product line since 1995 and consumed by many athletes. In the human studies, the preceding administration of VAAM at the dose of one eighth of our animal studies significantly lowered the respiratory quotient during the 60 min of cycling exercise [9], and the 12 weeks of VAAM administration at the same dose as above increased maximal oxygen uptake in the older women groups [10]. These results suggest that ingested VAAM may affect the metabolic state of multiple tissues, such as liver and adipose tissues, to facilitate energy supply to the locomotor system. However, there is no information on the effect of VAAM on the physiology of resting or freely moving animals. Here we present data indicating this type of regulation in multiple tissues at the gene transcriptional level.

## Results

### Characterization of genes regulated by VAAM administration in murine liver

**Fig. 1:**
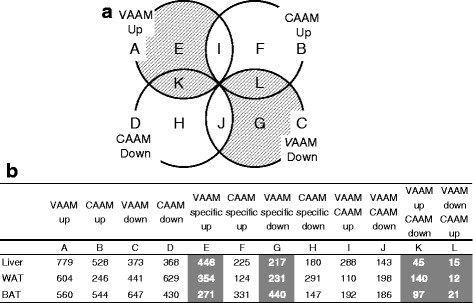
Effects of amino acid mixtures on tissue transcriptomes. **a** Number of genes regulated by VAAM and/or CAAM. VAAM Up or CAAM Up: up-regulated compared with the water group; VAAM Down or CAAM Down: down-regulated compared with the water group. **b** Number of regulated genes in the liver, WAT, and BAT

**Fig. 2:**
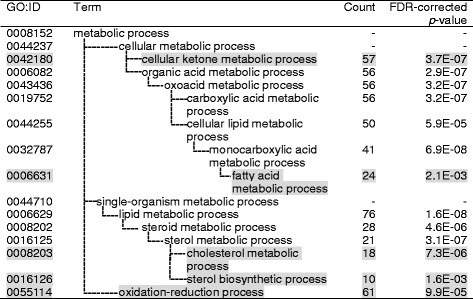
Gene ontology analysis of liver genes for which expression levels were affected by VAAM administration. 446 + 45 + 15 + 217 genes in Fig. 1b were used for analysis. GO terms located at the *bottom* of hierarchy are represented by *shaded text*

Mice under freely feeding and freely moving conditions were treated with VAAM, CAAM, or water once per day for 5 days and euthanized at 4 h after the last administration (Additional file 2: Figure S1). The energy contribution of VAAM and CAAM was 0.38 % of total food-derived energy/day (Additional file 1: Table S1). The biochemical analysis of mice sera showed no difference in terms of carbohydrates and lipids among the groups (Additional file 3: Table S2). We conducted DNA microarray analysis to investigate the physiological effect of VAAM administration. The differentially expressed genes (DEGs) were calculated by the statistic comparison between the water group and VAAM group or between the water group and CAAM group (see “Methods”). These DEGs were then set-theoretically subtracted or merged to extract DEGs specifically regulated by VAAM administration (Fig. 1). To elucidate the function of genes regulated by VAAM specifically, we applied GO analysis to the subset of the extracted genes. The group specifically up-regulated by VAAM administration was composed of 446 genes, 217 down-regulated genes, and 45 plus 15 genes inversely correlated among VAAM and CAAM, as shown in a Venn diagram (Fig. 1a, b). The GO terms attributed to these probe sets clearly indicate that the genes related to lipid metabolism are significantly enriched in these subsets (Fig. 2). It is also notable that 45 + 15 genes, which were oppositely regulated by VAAM and CAAM, respectively (Fig. 1b), include eight genes (cytochrome P450, family 7, subfamily b, polypeptide 1 (*Cyp7b1*), hydroxy-delta-5-steroid dehydrogenase, 3 beta- and steroid delta-isomerase 5 (*Hsd3b5*), phosphatidylcholine transfer protein (*Pctp*), sterol regulatory element binding transcription factor 1 (*Srebf1*), aldehyde dehydrogenase family 3, subfamily A2 (*Aldh3a2*), fatty acid binding protein 7, brain (*Fabp7*), 3-hydroxy-3-methylglutaryl-Coenzyme A synthase 1 (*Hmgcs1*), and retinol dehydrogenase 9 (*Rdh9*)) related to lipid metabolism; three genes (serine hydroxymethyltransferase 1 (soluble) (*Shmt*), solute carrier family 46, member 1 (*Slc46a1*), and thiopurine methyltransferase (*Tpmt*)) related to one-carbon metabolism; and one gene (pyruvate kinase liver and red blood cell (*Pklr*)) related to glycolysis (Additional file 4: Table S3).

### Regulation of liver metabolism by VAAM

**Fig. 3:**
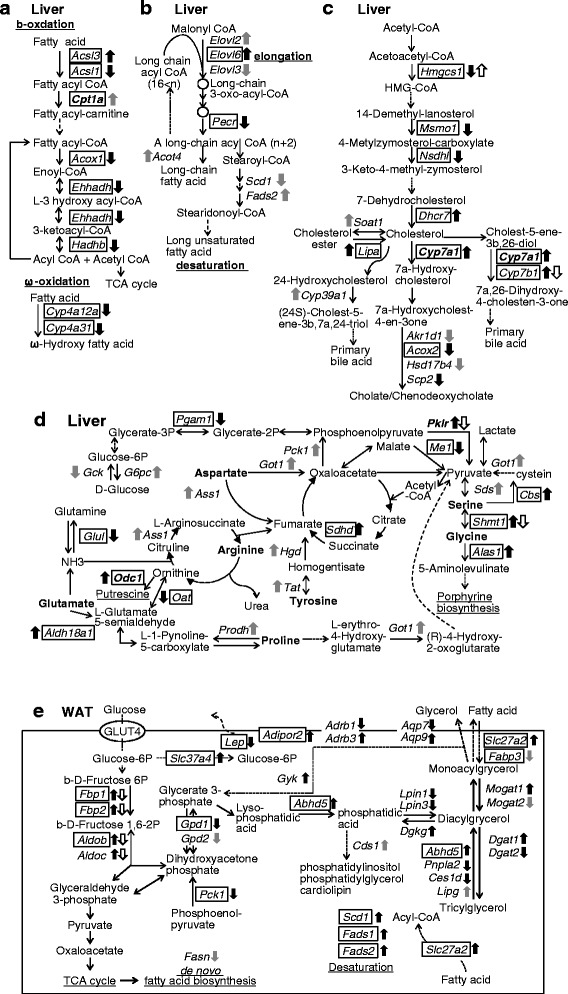
Metabolic function of VAAM-regulated genes in the liver and WAT. Gene symbols and their mode of regulation by VAAM (*black arrows*), by CAAM (*white arrows*), and by VAAM and CAAM (*gray arrows*) are represented in fatty acid oxidation (**a**), in fatty acid desaturation (**b**), in cholesterol metabolism (**c**), and in glycolysis, glucogenic amino acid metabolism, and the TCA cycle (**d**) in the liver. **e** Glucose and triacylglycerol metabolism of WAT. All gene symbols and their full names are listed in Additional file 5: Table S4

Mapping of the enriched genes to known metabolic pathways revealed a VAAM-specific regulation of lipid and amino acid metabolism, i.e., a down-regulation of beta- and omega-oxidation (Fig. 3a), the accumulation of middle chain fatty acid (Fig. 3b), the utilization of cholesterol stock for bile acid synthesis (Fig. 3c), and the utilization of glucogenic amino acids in the TCA cycle (Additional file 5: Table S4) (Fig. 3d). In particular, the oppositely regulated genes highlight the VAAM-specific effect on metabolic pathways; e.g., *Hmgcs1* and *Cyp7b1* in bile acid synthesis (Fig. 3a) and *Pklr* and *Shmt* in glucogenic amino acid utilization (Fig. 3d). We confirmed expression levels of *Elovl3* (elongation of very long chain fatty acids like 3), *Pecr* (peroxisomal trans-2-enoyl-CoA reductase), and *G6pc* (glucose-6-phosphatase, catalytic subunit) by quantitative PCR and found that *Elovl3* and *Pecr* were down-regulated in the VAAM group, while *G6pc* was up-regulated in the VAAM group as observed in DNA microarray analysis (Additional file 6: Figure S2).

### Response of adipose tissue transcriptomes to VAAM

**Fig. 4:**
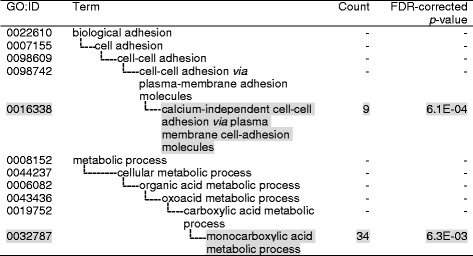
Gene ontology analysis of WAT genes for which expression levels were affected by VAAM administration. 354 + 140 + 12 + 231 genes in Fig. 1b were used for analysis. GO terms located at the *bottom* of hierarchy are represented by *shaded text*

Next, we applied a similar transcriptomic analysis to white and brown adipose tissues (WAT and BAT). In the case of WAT, the GO terms attributed to the VAAM-responsive genes (354 + 140 + 12 + 231 genes, Fig. 1a, b) were “calcium-independent cell-cell adhesion via plasma membrane cell-adhesion molecules” and “monocarboxylic acid metabolism process” (Fig. 4). Approximately, one half of the genes enriched in “monocarboxylic acid metabolism process” were related to lipid synthesis (17 out of 30, Additional file 7: Table S5). Mapping these genes in metabolic pathways revealed that VAAM up-regulates several genes for phospholipid synthesis from glucose and fatty acid (Fig. 3e). Four genes for enzymes of acylglycerol synthesis were oppositely regulated: up-regulated for monoacylglycerol O-acyltransferase 1 (*Mogat1*) and diacylglycerol O-acyltransferase 1 (*Dgat1*), and down-regulated for monoacylglycerol O-acyltransferase 2 (*Mogat2*) and diacylglycerol O-acyltransferase 2 (*Dgat2*). It is also notable that solute carrier family 37 (glucose-6-phosphate transporter), member 4 (*Slc37a4*), which facilitates glucose-6-phosphatase activity, is up-regulated.

**Fig. 5:**
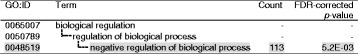
Gene ontology analysis of BAT genes for which expression levels were affected by VAAM administration. 271 + 97 + 21 + 440 genes in Fig. 1b were used for analysis. GO terms located at the *bottom* of hierarchy are represented by *shaded text*

GO analysis of VAAM-responsive genes in BAT (271 + 97 + 21 + 440 genes, Fig. 1a, b) resulted in the identification of the term “negative regulation of biological process” located at the bottom of the hierarchy (Fig. 5). These terms contain a smaller number of metabolic enzyme genes than is the case for liver and WAT, but those related to cellular signaling such as adiponectin receptor 2 (*Adipor2*), adenosine A1 receptor (*Adora1*), cyclin-dependent kinase inhibitor 1 (*Cdkn1*), glycogen synthase kinase 3b (*Gsk3b*), protein tyrosine phosphatase, non-receptor type (*Ptpn*), forkhead box O1 (*Foxo1*), insulin-like growth factor 1 (*Igf1*), lymphocyte protein tyrosine kinase (*Lck*), mitogen-activated protein kinase (*Mapk*), and regulator of G-protein signaling (*Rgs*) (Additional file 8: Table S6). This finding prompted us to search for an unknown upstream factor that may regulate the adipose tissue transcriptomes in relation to that of the liver.

### Search for upstream regulators common among liver and adipose tissues

We searched for Ingenuity Pathway Analysis (IPA) upstream regulators of VAAM-regulated genes starting with liver genes (446 + 217 genes), followed by comparison with the WAT genes (354 + 231 genes) and the BAT genes (271 + 440 genes), respectively. The liver transcriptome exhibited a total of 103 activated and 32 inhibited upstream regulators (absolute Z-scores >2.0, Table 1). Because some of these regulators have close functional relationships to each other, they were classified into the same columns (e.g., “isoproterenol”, “norepinephrine” as a beta-agonist, and “propranolol” as a beta-antagonist). Interestingly, there are obvious inter-tissue correlations of selected regulators, including “norepinephrine” (2.06 in WAT and 3.05 in BAT) and “progesterone” (Z-score of 2.50 in liver and 2.12 in WAT). Considering the functional similarity of these selected regulators, it is very possible that beta-adrenergic and TGF-related signals may be activated in these three tissues. It is also noteworthy that estrogen, glucagon, and gentamicin signals seemed to be activated in the liver and WAT and that I-kappa B, interleukins 5 and 6, insulin, PDGF, and the other signals were activated in BAT and WAT (Table 1). There was a discrepancy in the Z-score between “INS”, at −2.18 in the liver, and “Ins1”, at 2.23 in BAT. The mouse genome contains two genes for insulin (Ins1 and Ins2), each of which corresponds to “Ins1” and “INS” in the IPA database, respectively. It is possible that this discrepancy is due to functional differences between the Ins1 and Ins2 genes.

**Table 1:**
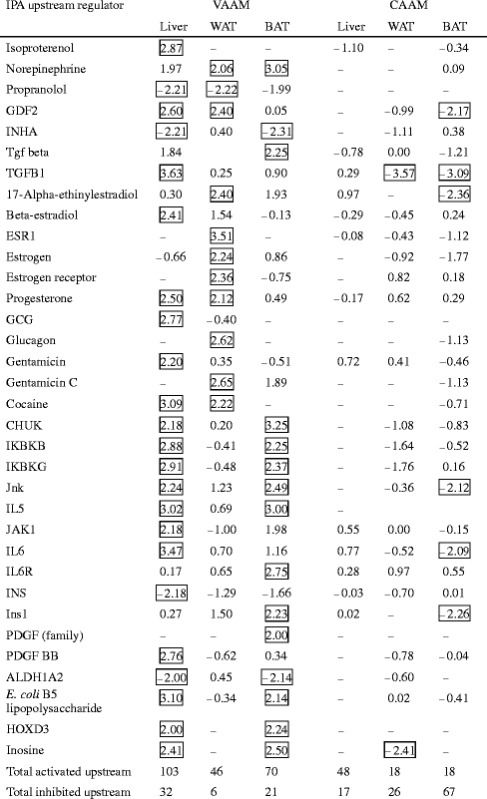
Correlation of IPA upstream regulators between the liver, WAT, and BAT transcriptomes

The values show the activation Z-score. The absolute Z-scores ≥2 are in squares

*ALDH1A2* aldehyde dehydrogenase 1 family, member A2, *CHUK* conserved helix-loop-helix ubiquitous kinase, *ESR1* estrogen receptor 1, *GCG* glucagon, *GDF2* growth differentiation factor 2, *HOXD3* homeobox D3, *IKBKB* inhibitor of kappa light polypeptide gene enhancer in B cells, kinase beta, *IKBKG* inhibitor of kappa light polypeptide gene enhancer in B cells, kinase gamma, *IL5* interleukin 5, *IL6* interleukin 6, *IL6R* interleukin 6 receptor, *INHA* inhibin, alpha, *INS* insulin, *Ins1* insulin I, *JAK1* Janus kinase 1, *PDGF* platelet-derived growth factor, *TGFB1* transforming growth factor, beta 1

## Discussion

In this study, we examined the non-nutritive effect of a specific amino acid mixture, VAAM, on the liver, WAT, and BAT transcriptomes in mice under freely moving conditions. From the gene ontology (GO) analysis of VAAM-regulated genes, it was predicted that VAAM may up-regulate sugar and lipid anabolism in these tissues. In addition, a comparative analysis of IPA upstream factors among these tissues suggested that estrogenic and glucagon signals were activated in the liver and WAT and that beta-adrenergic signaling was activated in the three tissues.

Our previous studies [6, 7] on VAAM were conducted using a mouse model under forced exercise conditions, where the glycogen storage might have been exhausted and the muscle might have continued to move with the help of a fatty acid supply from the adipose tissues. The major difference between the previous studies and the present study resides in whether or not moving conditions were present (forced swimming or free motion) and in the dosing condition (one shot or five shots). However, there are some correlations in the metabolic regulation among these studies. We observed a down-regulation of beta-oxidation and an up-regulation of glucogenic amino acid utilization in the liver (Fig. 3). The former may save the fatty acid pool in total and the latter makes it possible to supply amino acid-derived substrates to gluconeogenesis in response to exercise stimulus [11–13]. In the case of WAT, four enzyme genes for acylglycerol synthesis were regulated in a distinct manner: *Mogat1* and *Dgat1* were up-regulated, and *Mogat2* and *Dgat2* were down-regulated. Especially, the opposite regulation of *Dgat1* (up) and *2* (down) should be mentioned because *Dgat1* facilitates fatty acid/acylglycerol shuttling, and *Dgat2* is responsible for triacylglycerol accumulation [14]. This regulation may support fatty acid release to the serum. It is also possible that the up-regulation of *Slc37a4* may decrease lactate production by facilitating glucose regeneration from glucose-6-phosphate (Fig. 3e). The role of BAT in exercise performance is not clear because our experiments were performed at a moderate temperature, where no additional thermogenesis is required. However, it should be noted that the two catecholamine responsive genes, deiodinase, iodothyronine, type II (*Dio2*) and peroxisome proliferator-activated receptor gamma, coactivator 1 alpha (*Ppargc1a*, also known as *Pgc1a*), are up-regulated. These genes are reported to be induced in mice and rats exposed to cold temperature [15, 16]. In the moderate temperature condition as those we adopted, this kind of regulation may result in an increased basal metabolic rate under resting conditions [12, 17, 18]. In conclusion, the VAAM-dependent regulation of multiple tissue transcriptomes seems to be a tuning mechanism that makes the metabolic state resistant to more intense exercise. It would be valuable to examine the effect of middle term (approximately 1 week) pre-administration of VAAM on exercise performance in a human interactive study.

Whether VAAM acts on these tissues directly or indirectly is an open question. From the IPA upstream factor analysis, it was predicted that the three endocrine factors adrenaline, estrogen, and glucagon seemed to stimulate these tissues. In particular, adrenaline is the most plausible factor involved in VAAM signaling because several IPA terms related to adrenergic signals were detected in the three tissues (Table 1) and its increase in the mice sera was detected in a previous study [7]. Considering that a major source of adrenaline is the hypothalamus-sympathetic nerve-adrenal medullary (HSA) axis, we think it is highly possible that VAAM controls terminal tissues via this system. This assumption raises another question as to what type of mechanism transduces VAAM signals in the HSA axis. There are two supporting information. First is that VAAM’s effect depends on the overall ratio of amino acids (Additional file 1: Table S1), and second, the maximal effect of VAAM was obtained with the pretreatment of animals 30 min before the exercise. These considerations suggest that the sensing mechanism for VAAM might have a broad spectrum to amino acids and that the mechanism may reside in the gastrointestinal tract that faces the VAAM solution but not in metabolizing tissues nor locomotor systems. Accordingly, we assume that gastrointestinal chemosensory cells are one of the candidate targets of VAAM stimulus. Further investigation is needed to elucidate the VAAM-sensing and VAAM-transducing machinery.

## Conclusions

We found that VAAM ingestion had an effect on multiple tissue transcriptomes of freely moving mice. Utilization of glucogenic amino acids may have been activated in the liver. Fatty acid conversion into phospholipid, not to triacylglycerol, may have been stimulated in adipocytes contrasting that a little effect was observed in BAT. Analysis of upstream factors revealed that multiple hormonal signals were activated in the liver, WAT, and BAT. Our data provide some clues to understanding the role of VAAM in metabolic regulation.

## Methods

### Animals

Five-week-old male ddY mice (SLC Japan, Shizuoka, Japan) were housed in plastic cages with food (MF, Oriental yeast, Tokyo, Japan) and deionized water ad libitum in a temperature- and humidity-controlled room with a 12-h light/dark cycle (light 08:00–20:00; dark 20:00–08:00). The mice were weighed every day. All animal experimental protocols were approved by the Animal Use Committee of the Faculty of Agriculture at the University of Tokyo with approval number: P12-669.

### Amino acids

Glycine and all l-amino acids were purchased from Wako Pure Chemical Industries (Osaka, Japan). The amino acid mixtures VAAM and CAAM were prepared in the ratios described by Abe et al. [6] and were analyzed for amino acid composition using a Hitachi l-8500A amino acid analyzer (Hitachi High Technologies, Tokyo) (Additional file 1: Table S1). The concentrations of VAAM and CAAM were each adjusted to 1.8 %.

### Experimental procedure

The experimental schedule is shown in Additional file 2: Figure S1. The mice (*n* = 21) were acclimated to a laboratory environment for 1 week. They were divided randomly into three treatment groups: VAAM, CAAM, or water. Using a feeding tube, 1.8 % VAAM, 1.8 % CAAM, or water was orally administered five times once a day at 10:00AM. The dosage of VAAM, CAAM, or water was adjusted to 37.5 μL per gram of body weight as described previously [6]. On the day of last administration, the food was removed and the mice were moved to clean cages at 8:00. The last treatments began at 10:00. At 4 h after the last administration, the mice were euthanized by cervical dislocation and the blood, liver, WAT, and BAT were collected. The blood was separated to obtain the serum. Small hepatic pieces were immersed into RNAlater (Qiagen, Tokyo, Japan). The WAT and BAT were immediately frozen after extraction using liquid nitrogen. All samples were maintained at −80 °C until use. Two mice in CAAM group were excluded because of their morphological abnormality in the liver. Subsequently, the mice number of each group became *n* = 7 for the VAAM group, *n* = 5 for the CAAM group, and *n* = 7 for the water group.

### Measurement of serum biochemical parameters

Serum glucose, non-esterified fatty acid (NEFA), and triacylglycerol were enzymatically assayed using the glucose C II test Wako, the NEFA C test Wako, and the triglyceride E test Wako (Wako Pure Chemical Industries, Osaka, Japan), respectively. Total lipids, total ketone bodies, total cholesterol, HDL cholesterol and LDL cholesterol were assayed on Nagahama Life Science (Shiga, Japan). Each of the values is represented as the mean ± standard error of the mean (SEM). Significant differences (*p* ≤ 0.05) between the experimental groups were assessed by Tukey-Kramer comparison.

### DNA microarray assay

Total RNA was isolated from each hepatic, WAT, and BAT sample by TRIzol reagent (Invitrogen Japan, Tokyo, Japan) and purified using an RNeasy mini kit (Qiagen, Tokyo, Japan). The RNA Integrity Number (RIN) was estimated as an index of the quality of the total RNA using an Agilent 2100 Bioanalyzer (Agilent Technologies Japan, Tokyo, Japan). The values for RIN were over 8.3.

Then, amplified RNA (aRNA) was synthesized from 200 ng of purified total RNA, and biotinylated aRNA was obtained using a GeneChip® 3′IVT Express Kit (Affymetrix, Santa Clara, CA, USA). The aRNA was fragmented and hybridized to a GeneChip® Mouse Genome 430 2.0 Array (Affymetrix) for 16 h at 45 °C. The arrays were washed and stained with phycoerythrin using the GeneChip® Fluidics Station 450 (Affymetrix). The arrays were submitted to scanning on an Affymetrix GeneChip® Scanner 3000 7G (Affymetrix). The Affymetrix® GeneChip® Command Console® Software (Affymetrix) was used to make CEL files.

### DNA microarray data analysis

The CEL files were quantified by the distribution free weighted method (DFW) [19] using the statistical language R (2.7.1) (http://www.r-project.org/) [20] and Bioconductor (2.2) (http://www.bioconductor.org/) [21]. Hierarchical clustering was performed using the pvclust() function in R [22]. The rank products (RP) method was used to identify differentially expressed gene probe sets of the DFW-quantified data [23]. The probe sets with a false discovery rate (FDR) <0.05 were considered to be differentially expressed between two groups. RP was performed for a comparison of the water treatment group and the VAAM or CAAM treatment groups.

### Gene ontology analysis

The up- and down-regulated probe sets picked out at FDR <0.05 were functionally classified by the biological process in gene ontology (GO) with the Functional Annotation Tool of the Database for Annotation, Visualization, and Integrated Discovery (DAVID) [24, 25] and Quick GO (http://www.ebi.ac.uk/QuickGO/) [26]. EASE scores, which are modified Fisher’s exact test *p* values, were used to extract statistically overrepresented GO terms. GO terms with FDR-corrected *p* values <0.01 were regarded as significantly enriched.

### Pathway analysis

The differentially expressed genes were mapped on metabolic pathways in reference to the Kyoto Encyclopedia of Genes and Genomes (www.genome.ad.jp/kegg) [27].

### Upstream regulator analysis

Predicted upstream regulators from VAAM- and CAAM-specific data sets were analyzed using QIAGEN’s Ingenuity® Pathway Analysis (IPA®, QIAGEN Redwood City, www.qiagen.com/ingenuity). The activation Z-score was calculated as a measure of upstream regulator analysis. An absolute Z-score ≥2 was judged as significantly activated or inhibited, while an absolute Z-score ≤1.0 could not judge a direction. Common upstream regulators that were predicted to be activated or inhibited in the liver, WAT, and BAT were selected from the all upstream regulators list.

### Quantitative PCR analysis

Total RNA was prepared as described in “DNA microarray assay” section. Complementary DNA (cDNA) was synthesized with PrimeScript RT reagent kit (TaKaRa, Shiga, Japan) and subjected to PCR analysis using the following primer sets and SYBR Premix Ex Taq II kit (TaKaRa). The cDNA was amplified by 30 s at 95 °C followed by 40 cycles of incubation at 95 °C for 15 s and at 60 °C for 30 s in CFX96 real-time PCR detection system (Bio-Rad). The primers sets were as follows: *Elovl3* forward; 5′-TTCTCACGCGGGTTAAAAATGG-3′, *Elovl3* reverse; 5′- GAGCAACAGATAGACGACCAC-3′, *G6pc* forward; 5′-TTCAAGTGGATTCTGTTTGG-3′, *G6pc* reverse; 5′- AGATAGCAAGAGTAGAAGTGAC-3′, *Pecr* forward; 5′-GAAGGGATGGCATGCTGTGA-3′, *Pecr* reverse; 5′- TTGACAATCGACCCTCCGTG-3′, *beta-actin* forward; 5′-GATGTATGAAGGCTTTGGTC-3′, *beta-actin* reverse; 5′- TGTGCACTTTTATTGGTCTC-3′. Each cycle threshold (CT) value of an amplified fragment was compared with that of beta-actin and represented as a relative expression level. Values for VAAM or CAAM treatment group and water treatment group were examined for their significant difference using Dunnett’s test.

## Abbreviations

BAT, brown adipose tissue; CAAM, casein amino acid mixture; EAAs, essential amino acids; NEFA, non-esterified fatty acid; VAAM, *Vespa* amino acid mixture; WAT, white adipose tissue

## Additional files

Additional file 1: Table S1. Comparison of amino acid mixtures (Mol%). The amino acid mixtures VAAM and CAAM were analyzed for amino acid composition using an amino acid analyzer. (DOCX 36 kb) Additional file 2: Figure S1. Feeding schedule of amino acid mixtures. VAAM, CAAM or water was orally administered five times once a day using a feeding tube. Daily administration was performed at 10:00. On the day of last administration, the food was removed and the mice were moved to clean cages at 8:00. The last treatments began at 10:00. At 4 hours after the last administration, the mice were euthanized by cervical dislocation and blood, liver, WAT and BAT were collected. (PPTX 53 kb) Additional file 3: Table S2. Serum biomedical indices of mice administrated amino acid mixtures. The values are represented as the mean ± standard error of the mean (SEM). (DOCX 26 kb) Additional file 4: Table S3. The list of liver genes showing significant enrichment in the GO-terms located at the bottom of the hierarchy. VAAM-specific genes mapped on Figure 3 were represented by bold style. (XLSX 11 kb) Additional file 5: Table S4. Genes mapped on Fig. 3. (XLSX 13 kb) Additional file 6: Figure S2. The relative hepatic mRNA expression levels of *Elovl3*, *G6pc*, and *Pecr* in VAAM, CAAM or Water group. Quantitative PCR analysis were performed to validate the expression of metabolic genes, *Elovl3* (elongation of very long chain fatty acids like 3), *Pecr* (peroxisomal trans-2-enoyl-CoA reductase) and *G6pc* (glucose-6-phosphatase, catalytic subunit) in Water and VAAM or CAAM groups. *: p ≤ 0.05 by Dunnett’s test. (PPTX 69 kb) Additional file 7: Table S5. The list of WAT genes showing significant enrichment in the GO-terms located at the bottom of the hierarchy. VAAM-specific genes mapped on Figure 3 were represented by bold style. (XLSX 11 kb) Additional file 8: Table S6. The list of BAT genes showing significant enrichment in the GO-terms located at the bottom of the hierarchy. The list of BAT genes showing significant enrichment in the GO-terms located at the bottom of the hierarchy. (XLSX 12 kb)
