# Supplementary material for: Coordinated regulation of hepatic and adipose tissue transcriptomes by the oral administration of an amino acid mixture simulating the larval saliva of Vespa species
Source: Genes Nutr. 2016 Jul 11;11:21. doi: 10.1186/s12263-016-0534-2 (PMC4968451; doi:10.1186/s12263-016-0534-2)
Supplement: Additional file 1: Table S1. — Comparison of amino acid mixtures (Mol%). The amino acid mixtures VAAM and CAAM were analyzed for amino acid composition using an amino acid analyzer. (DOCX 36 kb) [file 12263_2016_534_MOESM1_ESM.docx]

Table S1 Composition of amino acid mixtures (Mol%)

| Amino acids | VAAM | CAAM | MF diet |
| --- | --- | --- | --- |
| Glycine | 19.1 | 4.5 | 9.4 |
| Alanine | 6.0 | 4.5 | 8.0 |
| Valine | 5.9 | 5.5 | 5.5 |
| Leucine | 6.2 | 8.5 | 7.9 |
| Isoleucine | 4.5 | 5.5 | 4.1 |
| Serine | 2.5 | 8.0 | 6.3 |
| Threonine | 7.2 | 2.5 | 4.5 |
| Cysteine | - | 0.4 | - |
| Cystine | - | - | 0.9 |
| Methionine | 0.5 | 2.5 | 1.8 |
| Aspartic acid | 0.2 | 7.5 | 9.6 |
| Glutamine | 3.2 | 19.6 | 16.2 |
| Arginine | 3.5 | 3.0 | 4.9 |
| Lysine | 8.6 | 7.0 | 5.1 |
| Histidine | 2.6 | 2.5 | 2.3 |
| Phenylalanine | 3.8 | 4.0 | 3.8 |
| Tyrosine | 6.0 | 5.0 | 2.2 |
| Tryptophan | 2.2 | 1.0 | 0.8 |
| Proline | 18.0 | 8.5 | 6.8 |
| Intake (g/ kg of body weight/ day) | 0.675 | 0.675 | 43.18 |
| Energy ratio to total intake (%) | 0.38 | 0.38 | 24.1 |
