# Supplementary material for: Coordinated regulation of hepatic and adipose tissue transcriptomes by the oral administration of an amino acid mixture simulating the larval saliva of Vespa species
Source: Genes Nutr. 2016 Jul 11;11:21. doi: 10.1186/s12263-016-0534-2 (PMC4968451; doi:10.1186/s12263-016-0534-2)
Supplement: Additional file 2: Figure S1. — Feeding schedule of amino acid mixtures. VAAM, CAAM or water was orally administered five times once a day using a feeding tube. Daily administration was performed at 10:00. On the day of last administration, the food was removed and the mice were moved to clean cages at 8:00. The last treatments began at 10:00. At 4 hours after the last administration, the mice were euthanized by cervical dislocation and blood, liver, WAT and BAT were collected. (PPTX 53 kb) [file 12263_2016_534_MOESM2_ESM.pptx]

## Slide 1
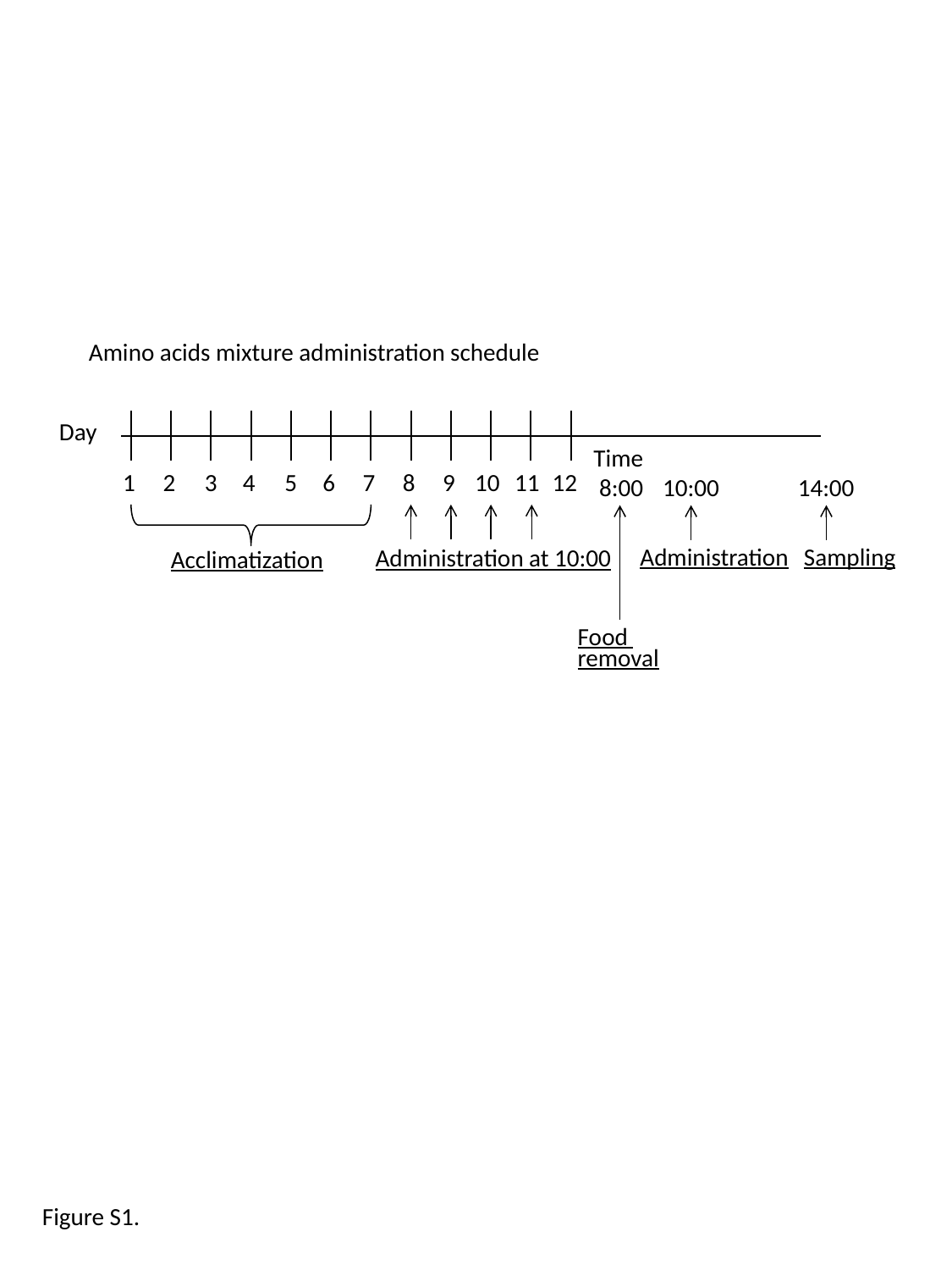

Amino acids mixture administration schedule
Day
1
2
3
4
5
6
7
8
9
10
11
12
Administration at 10:00
Acclimatization
Time
8:00
10:00
14:00
Administration
Sampling
Food
removal
Figure S1.
