# Supplementary material for: Coordinated regulation of hepatic and adipose tissue transcriptomes by the oral administration of an amino acid mixture simulating the larval saliva of Vespa species
Source: Genes Nutr. 2016 Jul 11;11:21. doi: 10.1186/s12263-016-0534-2 (PMC4968451; doi:10.1186/s12263-016-0534-2)
Supplement: Additional file 3: Table S2. — Serum biomedical indices of mice administrated amino acid mixtures. The values are represented as the mean ± standard error of the mean (SEM). (DOCX 26 kb) [file 12263_2016_534_MOESM3_ESM.docx]

| Table S2-Serum biochemical indices of mice administered amino acid mixtures | | | |
| --- | --- | --- | --- |
|  | Water (n=7) | VAAM (n=7) | CAAM (n=5) |
| Glucose (mg/dL) | 189 ± 11.5 | 187 ± 16.5 | 185 ± 6.30 |
| Total ketone body（μmol/L) | 434 ± 66.1 | 459 ± 113 | 625 ± 142 |
| Total lipid (mg/dL) | 491 ± 33.3 | 441 ± 15.2 | 483 ± 33.3 |
| Triglyceride (mg/dL) | 136 ± 13.0 | 110 ± 7.70 | 131 ± 9.50 |
| NEFA (mEq/L) | 1.30 ± 0.10 | 1.10 ± 0.10 | 1.20 ± 0.10 |
| Total Cholesterol (mg/dL) | 164 ± 11.3 | 156 ± 4.90 | 163 ± 12.0 |
| HDL cholestrerol (mg/dL) | 89.0 ± 5.70 | 85.3 ± 3.80 | 88.6 ± 6.50 |
| LDL cholesterol (mg/dL) | 13.1 ± 1.60 | 14.0 ± 2.00 | 11.6 ± 1.30 |
| Data are means ± SEM. | | | |
