# Supplementary material for: Coordinated regulation of hepatic and adipose tissue transcriptomes by the oral administration of an amino acid mixture simulating the larval saliva of Vespa species
Source: Genes Nutr. 2016 Jul 11;11:21. doi: 10.1186/s12263-016-0534-2 (PMC4968451; doi:10.1186/s12263-016-0534-2)
Supplement: Additional file 6: Figure S2. — The relative hepatic mRNA expression levels of Elovl3, G6pc, and Pecr in VAAM, CAAM or Water group. Quantitative PCR analysis were performed to validate the expression of metabolic genes, Elovl3 (elongation of very long chain fatty acids like 3), Pecr (peroxisomal trans-2-enoyl-CoA reductase) and G6pc (glucose-6-phosphatase, catalytic subunit) in Water and VAAM or CAAM groups. *: p ≤ 0.05 by Dunnett’s test. (PPTX 69 kb) [file 12263_2016_534_MOESM6_ESM.pptx]

## Slide 1
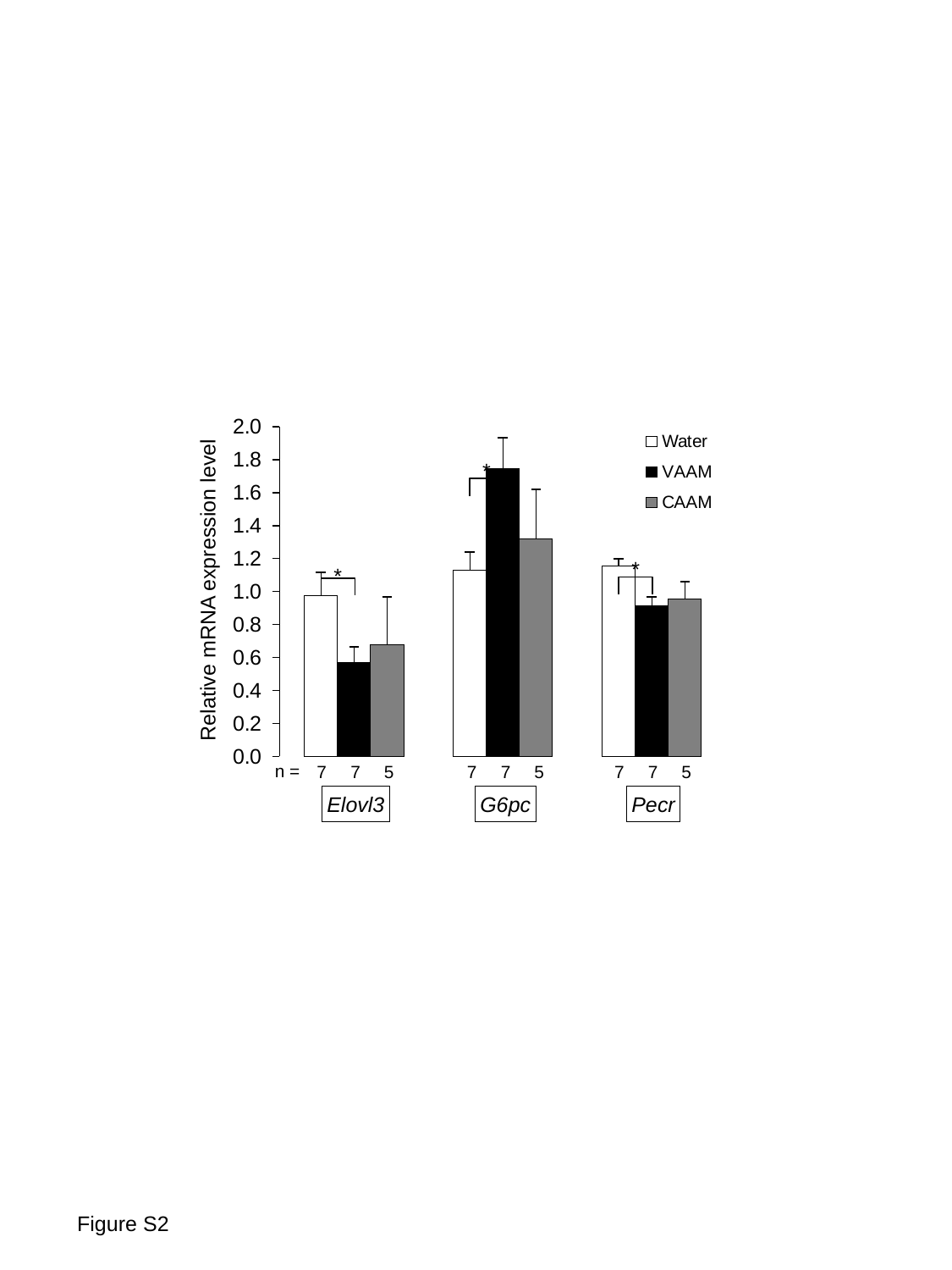

### Chart
| Category | Water | VAAM | CAAM |
|---|---|---|---|
| Elovl3 | 0.9752211 | 0.571822 | 0.6749822 |
| G6pc | 1.1279772857142858 | 1.7460982857142857 | 1.3196114 |
| Pecr | 1.1532197 | 0.9137066 | 0.9536899 |*
*
*
Relative mRNA expression level
n =
7
7
5
7
7
5
7
7
5
Elovl3
G6pc
Pecr
Figure S2
